# Suicide and mortality following self-harm in Culturally and Linguistically Diverse communities in Victoria, Australia: insights from a data linkage study

**DOI:** 10.3389/fpubh.2024.1256572

**Published:** 2024-03-27

**Authors:** Thi Thu Le Pham, Kerry S. O'Brien, Sara Liu, Katharine Gibson, Janneke Berecki-Gisolf

**Affiliations:** ^1^Victorian Injury Surveillance Unit, Monash University Accident Research Centre, Monash University, Clayton, VIC, Australia; ^2^School of Social Sciences, Monash University, Melbourne, VIC, Australia; ^3^Department of Health, Melbourne, VIC, Australia

**Keywords:** self-harm, suicide, mortality outcomes after self-harm, mental health, CALD, cultural backgrounds, multicultural, country of birth

## Abstract

**Background:**

While cultural backgrounds are well-documented to be relevant to intentional self-harm, little is known about how cultural and linguistically diverse (CALD) backgrounds affect mortality outcomes following self-harm.

**Aim:**

This study aimed to compare the risk of all-cause mortality and suicide after intentional hospital admissions for self-harm among people from CALD (vs. non-CALD) backgrounds.

**Method:**

Linked hospital and mortality data in Victoria, Australia, was used to assess suicide and all-cause death after hospital admissions for self-harm among patients aged 15+ years. All-cause death was identified by following up on 42,122 self-harm patients (hospitalized between 01 July 2007 and 30 June 2019) until death or 15 February 2021. Suicide death was evaluated in 16,928 self-harm inpatients (01 January 2013 and 31 December 2017) until death or 28 March 2018. Cox regression models were fitted to compare mortality outcomes in self-harm patients from CALD vs. non-CALD backgrounds.

**Outcomes:**

During the follow-up periods, 3,716 of 42,122 (8.8%) participants died by any cause (by 15 February 2021), and 304 of 16,928 (1.8%) people died by suicide (by 28 March 2018). Compared to the non-CALD group, CALD intentional self-harm inpatients had a 20% lower risk of all-cause mortality (HR: 0.8, 95% CI: 0.7–0.9) and a 30% lower risk of suicide (HR: 0.7, 95% CI: 049–0.97). Specifically, being from North Africa/Middle East and Asian backgrounds lowered the all-cause mortality risk; however, the suicide risk in Asians was as high as in non-CALD people.

**Conclusion:**

Overall, people from CALD backgrounds exhibited lower risks of all-cause mortality and suicide following hospital admission for self-harm compared to the non-CALD group. However, when comparing risks based on regions of birth, significant variations were observed. These findings underscore the importance of implementing culturally tailored background-specific suicide preventive actions. The study focussed on outcomes following hospital admission for self-harm and did not capture outcomes for cases of self-harm that did not result in hospital admission. This limits generalisability, as some CALD people might avoid accessing healthcare after self-harm due to cultural factors. Future research that not limited to hospital data is suggested to build on the results.

## Introduction

Intentional self-harm, such as self-poisoning or self-cutting with and without suicidal intent, is a serious public health problem in Australia (1). Intentional self-harm (hereinafter referred to as self-harm) is more common in female individuals, at younger ages, in Indigenous people, in separated and divorced people, and in people or communities with precarious employment situations (2). Mental health conditions, previous self-harm attempts, physical illness, substance abuse, social isolation, a lack of connectedness, racism, and discrimination are all risk factors for self-harm (2). However, religious affiliation and cultural norms are listed as possible protective factors for self-harm. These risks and protective factors for self-harm might be relevant to people from different cultural backgrounds.

Regarding adverse health outcomes after self-harm, research showed that people who engaged in self-harm might have an increased risk of premature death and self-harm reoccurrence (3–8). Research also showed a strong relationship between self-harm history and suicide (9): hospital-treated self-harm patients are at 30–200 times higher risk of suicide within 12 months of hospital discharge (10). In 2020, 3,139 Australians died from suicide, equivalent to nine suicide deaths every day (11). To help reduce the rate of suicide, effective prevention after self-harm is called for.

In the policy position statement of the Australian government on the CALD population (2021), Suicide Prevention Australia suggested to Parliament that it develop a suicide prevention plan that includes a section on preventing suicide in culturally and linguistically diverse (CALD) communities (12). However, little is known about potential suicide prevention strategies after hospital admission for self-harm among CALD people in Australia. There is evidence in England that the risk of suicide following self-harm among Black people was lower than that of white people, but when comparing South Asians to white people, the risk was not statistically different (at *p* = 0.05) (13). The variations in the risk of suicide following self-harm in different CALD groups suggest different targeted and tailored suicide prevention initiatives and allocations might be needed for different CALD groups based on cultural backgrounds.

Given that Australia is one of the most multicultural migrant countries, examining the mortality outcomes, including all-cause mortality and suicide following self-harm, among the CALD populations is necessary to inform suicide prevention strategies and priorities. This study used state-wide linked hospital data and mortality data for Victoria, Australia, which is a highly multicultural society. This allowed us to compare the outcomes in various CALD backgrounds by regions of birth and suggest specific culturally related suicide prevention messages and directions for future research in suicide prevention. More broadly, it provides key evidence supporting models for suicide prevention in other countries with rapidly increasing migration.

## Methodology

This data linkage study consists of two retrospective analyses to evaluate (1) death from any causes and (2) suicide death following hospital admission for self-harm among self-harm hospital inpatients aged 15+ years in Victoria, Australia. We used linked hospital and mortality data to compare mortality risks among self-harm patients in CALD groups with the non-CALD group.

### Data sources

#### Victorian admitted episodes dataset

Hospital admission cases were extracted from the VAED, which records all hospital admissions in public and private hospitals in the state of Victoria. Hospital inpatient data were used for sample selection and to determine pre-existing health conditions.

The VAED is a compilation of demographic, administrative, and clinical data on all admitted patient episodes of care provided by public and private hospitals, rehabilitation centers, extended care facilities, and day procedure centers in Victoria. The dataset is maintained by the Victorian Government Department of Health (DH) Health Data Standards and Systems (HDSS) unit for morbidity monitoring, case mix-based funding, and analysis purposes in accordance with several healthcare reporting agreements. Cases recorded on the VAED are coded to ICD-10-AM (Australian Modification of ICD-10): the WHO International Statistical Classification of Diseases and Related Health Problems, Tenth Revision, and Australian Modification (14).

#### Victorian death index

All deaths in Victoria are registered with the Registry of Births, Deaths, and Marriages. Usually, death registration is done by the funeral director. The Registry of Births, Deaths, and Marriages provided the VDI data to the Center for Victorian Data Linkage (CVDL) under a Memorandum of Understanding. The data were then provided to researchers after ethics approval and the data linkage application process. The database contains the most up-to-date information on all deaths reported to coroners. However, the cause of death is not available in this database and is only available in the *Cause of Death Unit Record Files*. The VDI was used as an indicator of death due to any cause.

#### Cause of death unit record files

The State and Territory Registrars of Births, Deaths, and Marriage and State Coroners provide data relating to deaths registered in Australia in a given reference year to the Australian Bureau of Statistics. The Australian Bureau of Statistics then codes the cause of death data using ICD-10. The COD dataset is administrative by nature and, as such, is useful for long-term monitoring of suicide deaths^1^.

### Data sources to identify pre-existing conditions that occurred 12 months before the index admission date

#### Victorian emergency minimum dataset

Emergency department (ED) presentation cases were extracted from the VEMD, which records ED presentations at *39 Victorian public hospitals* with 24-h emergency departments.

The VEMD comprises demographic, administrative, and clinical data detailing ED presentations at Victorian public hospitals with designated 24-h emergency departments. Data are coded according to the relevant VEMD User Manual published by the Department of Health (15).

The VEMD dataset was used to examine hospital-related outcomes (subsequent injury presentation; repeat ED presentation due to self-harm) and pre-existing hospital service use.

#### Clinical public mental health service contacts: Client Management Interface/Operational Data Store

The CMI/ODS data relates to clinical services that focus on the assessment and treatment of people with mental illness. These services are grouped by location into area-based mental health services and are managed by general health facilities, such as hospitals. There are reporting requirements for the Victorian Government's public mental health services using the CMI/ODS system. CMI/ODS comprises two systems. The Client Management Interface (CMI) is the local client information system used by each public mental health service. The Operational Data Store (ODS) manages a set of selected data items from each CMI. Data in the CMI includes demographic information, clinical status, including diagnosis, and service history for each person registered on the CMI/ODS system as receiving public clinical mental health services (16).

### Linkage process

Data linkage is a technique for identifying records in and across 30 different Victorian health and human services datasets that belong to the same person, family, place, or event in a way that protects individual privacy and can be used for analysis. Data linkage was conducted by CVDL, the Victorian Department of Health. The CVDL uses linkage identifiers to anonymously identify content and case variables relating to an individual across datasets (17).

The CVDL team extracted the data based on an agreed-upon technical assessment and research proposal designed by the research team. They provided the linked data to the researchers in de-identified format; identifiers were removed and dates were replaced with encrypted dates. Therefore, informed consent from participants was not needed.

### Sample selection

This study recruited participants using the same sample selection method as described in our previous work (18). Hospital admissions for self-harm included episodes where individuals engaged in self-harm with or without the intention to die. We extracted self-harm admissions from the VAED based on the International Classification of Diseases 10th Revision Australian Modification (ICD-10-AM). Admission records were selected if they contained a first external cause indicating self-harm (X60–X84) and a principal diagnosis that was either an injury (S00–T98) or a mental or behavioral disorder (F00–F99) (19).

**Figure 1:**
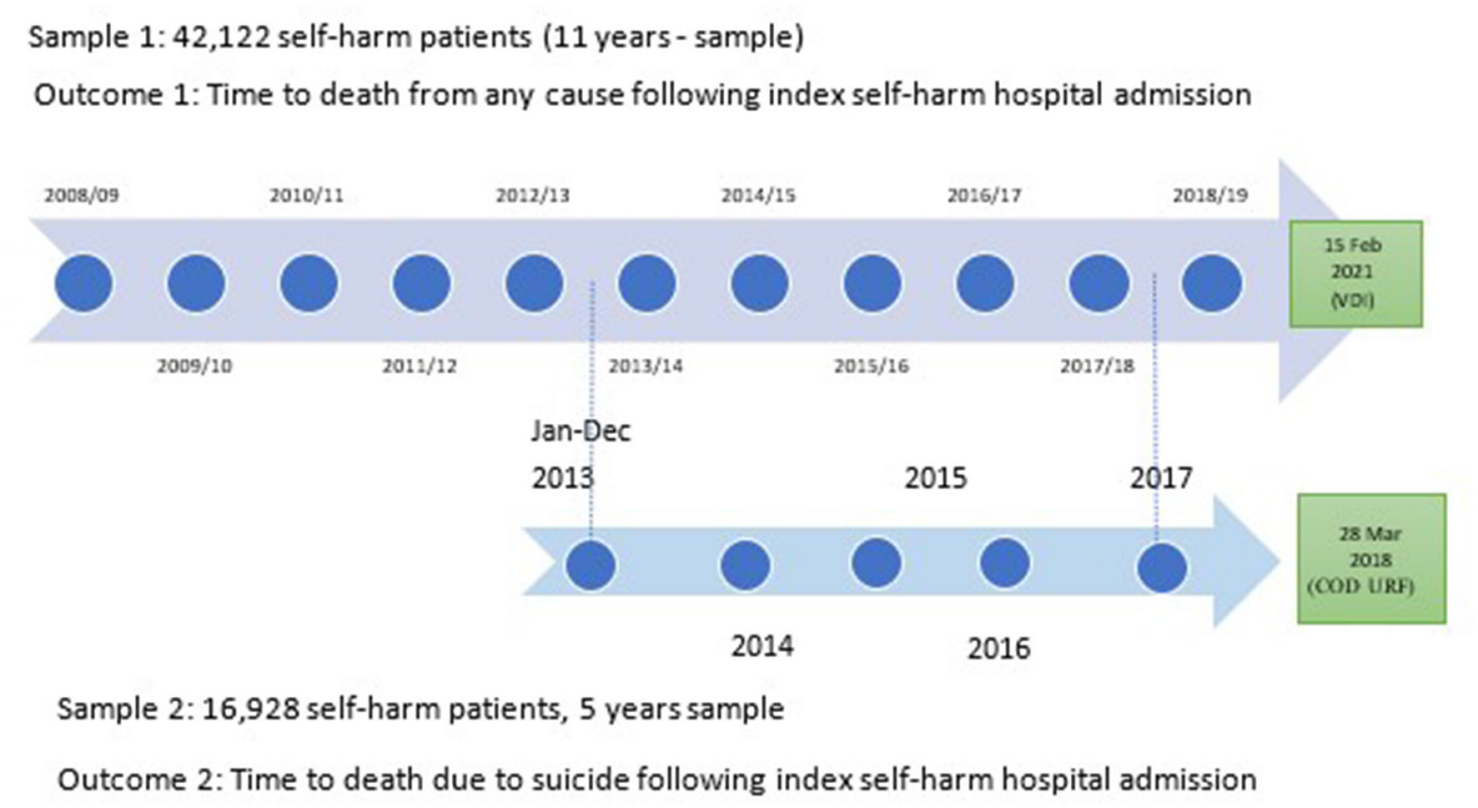
Schematic diagram of the data linkage periods to determine mortality outcomes.

Due to different data availabilities, two samples of participants were recruited to identify two outcomes. *Sample 1* included self-harm patients with their index hospital admissions for self-harm from 01 July 2007 to 30 June 2019 to identify all-cause deaths. *Sample 2* included those with index hospital admissions for self-harm from 01 January 2013 to 31 December 2017 to identify suicide deaths (Figure 1).

Grouping hospital admissions for self-harm episodes into periods of care was conducted to identify the incident episode, subsequent transfers, and statistical separations and statistical separations. This included instances where there was less than a 2-day gap between separation and readmission (18). Then, the index self-harm period of care was selected as the first (initial) self-harm period of care that occurred between the two timeframes mentioned above.

Participants were selected if they were 15 years and older and living in Victoria (regardless of their visa status). As participants with indeterminate or other sex were relatively rare within the samples (small cell counts), they were not included for confidentiality reasons. We excluded observations if patients had missing information on country of birth in VAED (< 1% of total participants).

### Outcomes

*Death from any cause after hospital admissions for self-harm* was identified by following Sample 1 until death or the data collection end date, which was 15 February 2021 (whichever came first). To flag the death outcome, Sample 1 data were linked with Victorian Death Index (VDI) data.

*Similarly, suicide death following hospital admissions for self-harm* was identified by following Sample 2 until death due to suicide or 28 March 2018 (whichever came first) in the linked COD URF data (Figure 1).

Suicide deaths were identified as having an ICD-10 underlying cause of death coded intentional self-harm (X60–X84). Undetermined intent death was not defined as suicide in this study. Including undetermined intent death in suicide is a controversial topic (20, 21). It is considered best practice to separate the analyses of intentional and undetermined intent deaths (22). However, as there were only 18 undetermined intent cases in Sample 2 (< 6 cases from the CALD group—Asia), we were not able to analyze these as a separate group due to low statistical power and data confidentiality. As a sensitivity analysis, we repeated the analysis, including undetermined intent deaths as suicide outcomes. The results show fairly similar findings (< 10% differences). Therefore, in this study, suicide deaths included those who died by intentional self-harm (X60–X84) only.

### CALD variables

The definition of CALD status of research participants and grouping CALD/non-CALD and sub-groups of CALD have been discussed elsewhere (18, 23).

#### CALD/non-CALD status of participants

The country of birth variable, as recorded in the VAED, was used to determine the CALD status of research participants. The CALD group included participants from non-English-speaking countries of birth (23). The CALD component “main language spoken at home” was not available in the data set and, therefore, was not used to define CALD status. Furthermore, although Indigenous people should be in a separate group (23) for data analysis due to their cultural diversity as well as having relatively high rates of self-harm compared to CALD and non-CALD groups (11), the small sample size of Indigenous peoples might affect the statistical power and data confidentiality of the research. For this reason, participants who self-identified as Indigenous peoples were grouped into CALD or non-CALD groups depending on their country of birth.

Research studies have shown that grouping all participants from CALD backgrounds into one group could make the group highly heterogeneous, and this could obscure important differences in self-harm (18, 24). We also divided the CALD group into nine subgroups based on region of birth (the Australian Bureau of Statistics (ABS)'s region classifications). Separate analyses comparing suicide/mortality risk in (1) the CALD group and (2) nine CALD subgroups with the non-CALD group were conducted to have more specific results.

#### CALD groups by nine regions of birth of participants (9 ROB/non-CALD)

- Oceania and Antarctica region, excluding Australia and New Zealand^1^;
- North-West Europe, excluding the United Kingdom (England, Wales, Scotland, and Northern Ireland) and the Republic of Ireland (see footnote 2);
- Southern and Eastern Europe;
- North Africa and the Middle East;
- South-East Asia;
- North-East Asia;
- Southern and Central Asia;
- Americas, excluding Canada and the United States of America (see footnote 2);
- Sub-Saharan Africa, excluding South Africa (see footnote 2).

### Demographic variables

Age group, sex, marital status, remoteness (Accessibility and Remoteness Index of Australia—ARIA+), and socioeconomic status (Socio-Economic Indexes for Areas—SEIFA) were extracted from VAED. The ARIA+ (25) and SEIFA were defined based on the residential addresses of patients as described elsewhere (18, 26). SEIFA was defined based on the Index of Relative Socio-Economic Advantage and Disadvantage, with state deciles based on statistical local areas. We regrouped participants into two groups of SEIFA, in which SEIFA Decile 1–5 indicated greater disadvantage, and Decile 6–10 indicated greater advantage.

### Factors relevant to self-harm admission

Mechanisms of self-harm (self-poisoning by pharmaceuticals, self-poisoning by other substances, self-harm by sharp object, and other means); place of injury (self-harm occurred at home, healthcare center, and other settings); comorbidity (27), any psychiatric disorder admission at the time of the index hospital admissions for self-harm (F00-F99), and alcohol use mentioned during index hospital admission (28) were extracted from hospital admission records. These factors were coded based on ICD-10-AM codes.

### Injury severity

Age-group stratified ICD-based injury severity scores (ICISS) based on the worst-injury method were used to calculate injury severity (29); serious injury was coded with an ICISS < 0.941, which means a survival probability of ≤ 94.1% (30, 31).

Pre-existing conditions within 12 months before the index admission date were identified based on ICD-10-AM codes in hospital datasets and by linking the samples with the Victorian Emergency Minimum Dataset (VEMD) and Clinical Public Mental Health Services (CMI/ODS) databases. These included *pre-existing hospital admissions due to self-harm* (VAED); *ED presentations due to self-harm*; *any admission due to mental illness* (ICD-10-AM coded as F00-F99 in VAED); and *mental health contacts before index admission* (any matched records found in linked clinical mental health service contact data, CMI/ODS).

### Statistical analysis

Data were analyzed using SAS version 14.1 and prepared using SPSS version 25 and STATA version 12.1. To describe and compare the characteristics of self-harm patients in the CALD group [and CALD subgroups by region of birth (ROB)] vs. the non-CALD group, percentages and 95% CIs of participants by socio-demographic characteristics, injury types, injury outcomes, self-harm and mental health history, and comorbidity were presented in Table 1.

**Table 1:** Characteristics of self-harm inpatients admitted to Victorian hospital 01 July 2007 and 30 June 2019 (Sample 1).

	**Non-CALD**				**CALD**			
	***N***	**%**	***CI (95%)***		***n***	**%**	***CI (95%)***	
**No of patients**	**36,503**	**86.7**	***86.3***	***87.0***	5,619	**13.3**	***13.0***	***13.7***
**Gender**								
Male	14,013	38.4	*37.9*	*38.9*	2,078	37.0	*35.7*	*38.2*
Female	22,490	61.6	*61.1*	*62.1*	3,541	63.0	*61.8*	*64.3*
**Age group**								
15–24	12,456	34.1	*33.6*	*34.6*	1,230	21.9	*20.8*	*23.0*
25–34	7,152	19.6	*19.2*	*20.0*	1,296	23.1	*22.0*	*24.2*
35–44	6,892	18.9	*18.5*	*19.3*	976	17.4	*16.4*	*18.4*
45–54	5,382	14.7	*14.4*	*15.1*	764	13.6	*12.7*	*14.5*
55–64	2,542	7.0	*6.7*	*7.2*	560	10.0	*9.2*	*10.7*
65+	1,846	5.1	*4.8*	*5.3*	785	14.0	*13.1*	*14.9*
**Accessibility and Remoteness Index of Australia (ARIA**+**)**								
Major Cities of Victoria, Australia	20,713	56.7	*56.2*	*57.3*	4,414	78.6	*77.5*	*79.6*
Inner Regional of Victoria, Australia	12,467	34.2	*33.7*	*34.6*	915	16.3	*15.3*	*17.2*
Outer Regional of Victoria, Australia	2,911	8.0	*7.7*	*8.3*	92	1.6	*1.3*	*2.0*
**Socio-Economic Indexes for Areas (SEIFA)**								
Decile 1–5 (greater disadvantage)	11,049	30.3	*29.8*	*30.7*	1,377	24.5	*23.4*	*25.6*
Decile 6–10 (greater advantage)	25,048	68.6	*68.1*	*69.1*	4,044	72.0	*70.8*	*73.1*
**Marital status**								
Never married	22,613	61.9	*61.5*	*62.4*	2,143	38.1	*36.9*	*39.4*
Widowed/divorced/separated	4,123	11.3	*11.0*	*11.6*	890	15.8	*14.9*	*16.8*
Currently married/defacto	8,896	24.4	*23.9*	*24.8*	2,483	44.2	*42.9*	*45.5*
Not stated/inadequately described	871	2.4	*2.2*	*2.5*	103	1.8	*1.5*	*2.2*
**Mechanism of self-harm**								
Poisoning- pharmaceuticals	28,679	78.6	*78.1*	*79.0*	4,260	75.8	*74.7*	*76.9*
Poisoning other substances	1,766	4.8	*4.6*	*5.1*	454	8.1	*7.4*	*8.8*
Sharp object	4,088	11.2	*10.9*	*11.5*	594	10.6	*9.8*	*11.4*
Other means	1,970	5.4	*5.2*	*5.6*	311	5.5	*4.9*	*6.1*
**Place of injury occurred**								
Home	17,478	47.9	*47.4*	*48.4*	2,958	52.6	*51.3*	*53.9*
Health care center	2,377	6.5	*6.3*	*6.8*	247	4.4	*3.9*	*4.9*
Other settings	2,403	6.6	*6.3*	*6.8*	344	6.1	*5.5*	*6.7*
Missing	14,245	39.0	*38.5*	*39.5*	2,070	36.8	*35.6*	*38.1*
**Length of stay in hospital**								
< 2 days	22,043	60.4	*59.9*	*60.9*	3,231	57.5	*56.2*	*58.8*
2–7 days	8,739	23.9	*23.5*	*24.4*	1,331	23.7	*22.6*	*24.8*
8–30 days	4,355	11.9	*11.6*	*12.3*	787	14.0	*13.1*	*14.9*
31+ days	1,366	3.7	*3.5*	*3.9*	270	4.8	*4.2*	*5.4*
**Serious injury**	355	1.0	*0.9*	*1.1*	140	2.5	*2.1*	*2.9*
**Comorbidity**	2,159	5.9	*5.7*	*6.2*	522	9.3	*8.5*	*10.0*
**Pre-existing conditions**								
hospital admissions for self-harm history	587	1.6	*1.5*	*1.7*	43	0.8	*0.5*	*1.0*
Self-harm ED presentation history	2,905	8.0	*7.7*	*8.2*	208	3.7	*3.2*	*4.2*
Mental health disorder history	9,020	24.7	*24.3*	*25.2*	919	16.4	*15.4*	*17.3*
Clinical mental health contact history	11,435	31.3	*30.9*	*31.8*	1,050	18.7	*17.7*	*19.7*
**Mental health disorder in index admission**	19,571	53.6	*53.1*	*54.1*	2,889	51.4	*50.1*	*52.7*

Full data table by regions of birth in the Supplementary material.

The occurrence of outcomes and (where relevant) 95% CI were calculated and presented in **Tables 3**, **5**. For survival analyses, time-to-death was calculated as the number of days from the index episode of self-harm to the fixed end date of follow-up (censored cases, for those who survived until then), or until the date of death if this preceded the end of the follow-up interval.

Various Cox regression models were fitted to estimate the effects of CALD backgrounds on mortality outcomes. Model 1 is the univariable model, and the impacts of possible confounders (highlighted in Table 1) were tested in sequentially adjusted models: *model 2*—adjusted for gender and age; *model 3*—marital status and geography were added; *model 4*—mechanisms of self-harm, injury severity, comorbidity, and mental health illness during the index admissions were added; and *model 5*—*fully adjusted model*, pre-existing health service use due to self-harm and mental illnesses were added. Hazard ratios with 95% confidence intervals were calculated for all models.

### Ethical consideration

The Monash University Human Research Ethics Committee provided the requested ethical approval for this research (Project ID 27360).

## Results

There were 42,528 individuals hospitalized due to intentional self-harm in Victoria between 01 July 2007 and 30 June 2019 and selected for the study; among them, the country of birth was missing for 401 individuals; a further five people had death records in VDI where the death dates were earlier than the admission dates. They were excluded from the study, leaving the remaining Sample 1 of 42,122 people, and similarly, 16,928 people were included in Sample 2. Of these, 13.3% were from culturally diverse backgrounds: Southern and Eastern Europe (SEE) was the biggest CALD subgroup by ROB (accounted for 3.0% of Sample 1), followed by South-East Asia (2.2%). The characteristics of participants in Sample 1 are presented in Table 1.

Participants in both CALD and non-CALD groups were predominantly female individuals, living in major Victorian cities, and from higher SEIFA. CALD self-harm patients were older and more likely to be married/de facto than their non-CALD counterparts.

The most common self-harm method of choice in both CALD and non-CALD groups was self-poisoning by pharmaceuticals. Poisoning by other substances was more common among those from CALD backgrounds than non-CALD people.

During the index hospital admissions for self-harm, CALD people were less commonly admitted with mental health disorders than the non-CALD group, but more CALD patients were admitted with serious injuries or having at least one comorbidity than their non-CALD counterparts.

Pre-existing conditions, including hospital-treated self-harm, hospital-treated mental health, and clinical mental health service contact 12 months prior to index hospital admissions for self-harm, were less common in CALD people than their non-CALD counterparts (Table 1).

These characteristics of participants in Sample 1 were consistent with those in Sample 2.

### All-cause mortality risk following self-harm (Sample 1)

By the end of the follow-up on 15 February 2021, 3,716/42,122 people in Sample 1 (self-harm patients with their index hospital admissions for self-harm from 01 July 2007 to 30 June 2019) had died by any cause (8.8%), equivalent to an incidence rate of 13.2/1000 (CI: 12.8–13.6) person-years (Table 2).

**Table 2:** Incidence rates of all-cause mortality and suicide by CALD and non-CALD groups.

	**Deaths**	**Person-years at risk**	**IR (/1,000 person-years)**	***95% CI***	
**All-cause mortality (Sample 1)**					
Non-CALD	3,162	245,070	12.9	*12.5*	*13.3*
CALD	554	36,779	15.1	*13.8*	*16.3*
Oceania and Antarctica^*^	(0–6)^†^	985	^†^	*0.6*	*9.5*
North-West Europe^*^	93	3,367	27.6	*22.1*	*33.2*
Southern and Eastern Europe	293	7,959	36.8	*32.7*	*40.9*
North Africa and the Middle East	38	5,572	6.8	*4.7*	*9.0*
South-East Asia	38	6,438	5.9	*4.0*	*7.8*
North-East Asia	21	2,987	7.0	*4.0*	*10.0*
Southern and Central Asia	41	6,567	6.2	*4.3*	*8.1*
Americas^*^	13	1,269	10.2	*4.7*	*15.8*
Sub-Saharan Africa^*^	(10–20)^‡^	1,635	^‡^	*3.2*	*11.5*
**Total**	**3,716**	**281,850**	**13.2**	***12.8***	***13.6***
**Suicide mortality (Sample 2)**					
Non-CALD	304	76,324	4.0	*3.5*	*4.4*
CALD	40	11,922	3.4	*2.3*	*4.4*
Europe^*^	24	3,383	7.1	*4.3*	*9.9*
Asia	(10–20)^‡^	5,435	^‡^	*0.8*	*3.2*
Others^**^	(0–6)^†^	3,104	^†^	*0.2*	*3.0*
**Total**	**344**	**88,245**	**3.9**	***3.5***	***4.3***

^*^Persons/POC of those who were born in English-speaking countries were excluded from the relevant CALD groups by ROB, and they were included in the non-CALD group.

^**^Others: Oceania and Antarctica; North Africa and the Middle East, Americas, Sub-Saharan Africa.

^†^Range was reported instead of the absolute number because cell values were < 6.

^‡^Other cells in the same row and/or column may also be suppressed and reported with “Range^‡^” to maintain confidentiality.

Table 3 presents the results of Cox's proportional hazards regression models to examine the risk factors of all-cause mortality after hospital admissions for self-harm. The results showed that all-cause deaths were more likely to be male, of older age, and living in major cities in Victoria. Those who had a serious injury or comorbidity at the index hospital admissions for self-harm were at higher risk of all-cause mortality.

**Table 3:** Risk of death due to all causes among self-harm patients admitted to hospital from 01 July 2007 and 30 June 2019 (Sample 1), Cox's proportional hazards regression models: univariate, partly adjusted and fully adjusted models.

	**Death by any causes**					**Model 1**			**Model 2**			**Model 3**			**Model 4**			**Model 5**		
	**Total**	**Death**	***%***	***CI 95%***		***HR***	***95% CI***		**HR**	**95% CI**		**HR**	**95% CI**		**HR**	**95% CI**		**HR**	**95% CI**	
**CALD by regions of birth**																				
**Non-CALD**	**36,503**	**3,162**	***8.7***	***8.4***	***9.0***	**R**			**R**			***R***			***R***			***R***		
**CALD**	**5,619**	**554**	***9.9***	***9.5***	***10.2***	**1.2** ^§^	***1.1***	***1.3***	**0.8** ^§^	***0.7***	***0.9***	**0.8** ^§^	***0.7***	***0.8***	**0.8** ^§^	***0.7***	***0.8***	**0.8** ^§^	***0.7***	***0.9***
Oceania and Antarctica^*^	146	(0–6)^†^	^†^	*0.5*	*6.4*	0.4^§^	*0.2*	*1.0*	*0.4*	*0.2*	*1.1*	*0.5*	*0.2*	*1.1*	*0.4*	*0.2*	*1.0*	0.4^§^	*0.2*	*1.0*
North-West Europe^*^	529	93	*17.6*	*14.3*	*20.8*	2.2^§^	*1.8*	*2.7*	*0.9*	*0.8*	*1.2*	*0.9*	*0.8*	*1.2*	*0.9*	*0.8*	*1.2*	*1.0*	*0.8*	*1.2*
Southern and Eastern Europe	1,249	293	*23.5*	*21.1*	*25.8*	2.8^§^	*2.5*	*3.2*	*1.1*	*0.9*	*1.2*	*1.1*	*1.0*	*1.2*	*1.0*	*0.9*	*1.1*	*1.0*	*0.9*	*1.2*
North Africa and the Middle East	821	38	*4.6*	*3.2*	*6.1*	0.5^§^	*0.4*	*0.7*	0.5^§^	*0.4*	*0.7*	0.5^§^	*0.4*	*0.7*	0.5^§^	*0.4*	*0.7*	0.5^§^	*0.4*	*0.7*
South-East Asia	935	38	*4.1*	*2.8*	*5.3*	0.5^§^	*0.3*	*0.6*	0.5^§^	*0.3*	*0.7*	0.5^§^	*0.3*	*0.7*	0.4^§^	*0.3*	*0.6*	0.5^§^	*0.3*	*0.7*
North-East Asia	472	21	*4.4*	*2.6*	*6.3*	0.5^§^	*0.4*	*0.9*	0.6^§^	*0.4*	*0.9*	0.6^§^	*0.4*	*0.9*	0.5^§^	*0.3*	*0.8*	0.6^§^	*0.4*	*0.9*
Southern and Central Asia	1,045	41	*3.9*	*2.7*	*5.1*	0.5^§^	*0.4*	*0.7*	0.6^§^	*0.4*	*0.8*	0.6^§^	*0.5*	*0.9*	0.6^§^	*0.4*	*0.8*	0.6^§^	*0.5*	*0.9*
Americas^*^	177	13	*7.3*	*3.5*	*11.2*	*0.8*	*0.5*	*1.4*	*0.7*	*0.4*	*1.1*	*0.7*	*0.4*	*1.1*	*0.7*	*0.4*	*1.2*	*0.7*	*0.4*	*1.3*
Sub-Saharan Africa^*^	245	(10–20)^‡^	^‡^	*2.2*	*7.6*	*0.6*	*0.3*	*1.1*	0.5^§^	*0.3*	*0.9*	0.5^§^	*0.3*	*0.9*	0.6^§^	*0.3*	*1.0*	0.6^§^	*0.3*	*1.0*
**Sex**																				
Male	16,091	2,016	*12.5*	*12.0*	*13.0*				1.8^§^	*1.6*	*1.9*	1.8^§^	*1.6*	*1.8*	1.6^§^	*1.5*	*1.7*	1.6^§^	*1.5*	*1.7*
Female	26,031	1,700	*6.5*	*6.2*	*6.8*				*R*			*R*			*R*			*R*		
**Age groups**																				
15–24	13,686	330	*2.4*	*2.2*	*2.7*				*R*			*R*			*R*			*R*		
25–34	8,448	496	*5.9*	*5.4*	*6.4*				2.3^§^	*2.0*	*2.6*	2.4^§^	*2.1*	*2.8*	2.3^§^	*2.0*	*2.7*	2.2^§^	*1.9*	*2.6*
35–44	7,868	663	*8.4*	*7.8*	*9.0*				3.1^§^	*2.7*	*3.5*	3.4^§^	*3.0*	*4.0*	3.3^§^	*2.8*	*3.7*	3.1^§^	*2.7*	*3.5*
45–54	6,146	688	*11.2*	*10.4*	*12.0*				4.4^§^	*3.9*	*5.0*	5.0^§^	*4.3*	*5.7*	4.5^§^	*3.9*	*5.2*	4.2^§^	*3.7*	*4.9*
55–64	3,102	495	*16.0*	*14.7*	*17.2*				6.5^§^	*5.6*	*7.5*	7.5^§^	*6.4*	*8.7*	6.3^§^	*5.4*	*7.4*	6.1^§^	*5.2*	*7.1*
65+	2,631	1,038	*39.5*	*37.6*	*41.3*				19.8^§^	*17.5*	*22.2*	23.1^§^	*20.0*	*27.0*	16.8^§^	*14.5*	*19.6*	17.0^§^	*14.7*	*20.0*
**Accessibility and Remoteness Index of Australia (ARIA**+**)**																				
Major Cities of Victoria, Australia	25,127	2,236	*8.9*	*8.5*	*9.3*							*R*			*R*			*R*		
Inner Regional of Victoria, Australia	13,382	1,227	*9.2*	*8.7*	*9.7*							*1.0*	*0.9*	*1.1*	*1.0*	*0.9*	*1.1*	*1.0*	*0.9*	*1.1*
Outer Regional of Victoria, Australia	3,003	232	*7.7*	*6.8*	*8.7*							0.8^§^	*0.9*	*0.7*	0.8^§^	*0.7*	*0.9*	0.8^§^	*0.7*	*0.9*
**Socio-Economic Indexes for Areas (SEIFA)**																				
Decile 1–5 (greater disadvantage)	12,426	1,076	*8.7*	*8.2*	*9.2*							*1.0*	*0.9*	*1.1*	*1.0*	*0.9*	*1.1*	*1.0*	*0.9*	*1.1*
Decile 6–10 (greater advantage)	29,092	2,619	*9.0*	*8.7*	*9.3*							*R*			*R*			*R*		
**Marital status**																				
Never married	24,756	1,510	*6.1*	*5.8*	*6.4*							*R*			*R*			*R*		
Widowed/divorced/separated	5,013	905	*18.1*	*17.0*	*19.1*							*1.0*	*0.9*	*1.1*	*1.0*	*0.9*	*1.1*	*1.0*	*0.9*	*1.1*
Currently married/defacto	11,379	1,206	*10.6*	*10.0*	*11.2*							0.7^§^	*0.6*	*0.8*	0.7^§^	*0.7*	*0.8*	0.8^§^	*0.7*	*0.8*
Not stated/inadequately described	974	95	*9.8*	*7.9*	*11.6*							0.8^§^	*0.6*	*1.0*	0.7^§^	*0.6*	*0.9*	0.8^§^	*0.6*	*1.0*
**Mechanisms of self-harm injury**																				
Poisoning- pharmaceuticals	32,939	2,726	*8.3*	*8.0*	*8.6*										*R*			*R*		
Poisoning other substances	2,220	256	*11.5*	*10.2*	*12.9*										*1.1*	*1.0*	*1.3*	*1.1*	*1.0*	*1.3*
Sharp object	4,682	424	*9.1*	*8.2*	*9.9*										*1.0*	*0.9*	*1.1*	*1.0*	*0.9*	*1.1*
Other means	2,281	310	*13.6*	*12.2*	*15.0*										1.5^§^	*1.3*	*1.7*	1.5^§^	*1.3*	*1.7*
**Place of self-harm**																				
Home	20,436	1,996	*9.8*	*9.4*	*10.2*										*R*			*R*		
Health care center	2,624	218	*8.3*	*7.3*	*9.4*										*0.9*	*0.8*	*1.0*	0.8^§^	*0.7*	*0.9*
Other settings	2,747	342	*12.4*	*11.2*	*13.7*										*1.1*	*1.0*	*1.2*	*1.1*	*1.0*	*1.2*
Missing	16,315	1,160	*7.1*	*6.7*	*7.5*										0.9^§^	*0.8*	*1.0*	0.9^§^	*0.8*	*1.0*
**Length of stay**																				
< 2 days	25,274	1,489	*5.9*	*5.6*	*6.2*										*R*			*R*		
2–7 days	10,070	1,132	*11.2*	*10.6*	*11.9*										1.5^§^	*1.4*	*1.6*	1.5^§^	*1.4*	*1.6*
8–30 days	5,142	780	*15.2*	*14.2*	*16.1*										1.6^§^	*1.4*	*1.7*	1.5^§^	*1.3*	*1.6*
31+ days	1,636	315	*19.3*	*17.3*	*21.2*										1.4^§^	*1.2*	*1.6*	1.2^§^	*1.1*	*1.4*
**Serious injury**																				
Yes	495	195	*39.4*	*35.1*	*43.7*										1.5^§^	*1.3*	*1.8*	1.6^§^	*1.3*	*1.8*
No	41,264	3,504	*8.5*	*8.2*	*8.8*										*R*			*R*		
**Any comorbidity CHARLSON**																				
Yes	2,681	663	*24.7*	*23.1*	*26.4*										2.0^§^	*1.9*	*2.2*	2.0^§^	*1.8*	*2.2*
No	39,441	3,053	*7.7*	*7.5*	*8.0*										*R*			*R*		
**Any mental health disorder**																				
Yes	22,460	2,205	*9.8*	*9.4*	*10.2*										0.8^§^	*0.8*	*0.9*	0.8^§^	*0.7*	*0.8*
No	19,662	1,511	*7.7*	*7.3*	*8.1*										*R*			*R*		
**Pre-existing (last 12 months before index self-harm admission)**																				
**Hospital admission due to self-harm**																				
Yes	630	89	*14.1*	*11.4*	*16.8*													*1.0*	*0.8*	*1.2*
No	41,492	3,627	*8.7*	*8.5*	*9.0*													*R*		
**ED presentation due to self-harm (last 12 months)**																				
Yes	3,113	298	*9.6*	*8.5*	*10.6*													1.1^§^	*1.0*	*1.3*
No	39,009	3,418	*8.8*	*8.5*	*9.0*													*R*		
**Hospital admission due to (all F codes)**																				
Yes	9,939	1,394	*14.0*	*13.3*	*14.7*													1.7^§^	*1.6*	*1.8*
No	32,183	2,322	*7.2*	*6.9*	*7.5*													*R*		
**Clinical mental health service contact (CMI-ODS)**																				
Yes	12,485	1,359	*10.9*	*10.3*	*11.4*													1.2^§^	*1.1*	*1.3*
No	29,637	2,357	*8.0*	*7.6*	*8.3*													*R*		

Death from all causes was identified by following up the 42,122 self-harm patients (who were admitted to hospital from 01 July 2007 and 30 June 2019) until death or 15 Feb 2021 (whichever comes first).

^*^Persons/POC of those who were born in English-speaking countries were excluded from the relevant CALD groups by ROB, and they were included in the non-CALD group.

^**^Other regions included Oceania and Antarctica; North Africa and the Middle East; Americas; Sub-Saharan Africa.

^†^Range was reported instead of the absolute number because cell values were < 6.

^‡^Other cells in the same row and/or column may also be suppressed and reported with “Range^‡^” to maintain confidentiality.

^§^P-value < 0.05.

Regarding the relationship between health conditions and all-cause death after self-harm, Table 3 shows that the risk was higher among those with pre-existing health conditions of self-harm ED presentations or mental illnesses (than those without the conditions), but lower in patients currently diagnosed with mental illnesses during index hospital admissions for self-harm (than those without; Table 3).

#### Comparisons of all-cause mortality risks following index hospital admissions for self-harm among CALD vs. non-CALD

When grouping all CALD people together, CALD people had a higher incidence of suicide death than the non-CALD group [15.1 [CI: 13.8–16.3] vs. 12.9 [CI: 12.5–13.3] person-years]. The rate was highest in SEE [36.8 [CI: 32.7–40.9]] and lowest in Oceania and Antarctica (Table 2).

In Table 3, the univariable model (Model 1) showed that CALD people (as one group) were at higher risk of all-cause mortality than their non-CALD counterparts, especially those from SEE and North-West Europe (when broken down by regions of birth). However, in the multivariable models (fully adjusted for demographic, injury-related variables, and pre-existing health service use), we found opposite results: those from a CALD background were *20% less likely to die* within the follow-up time than the non-CALD group. Specifically, being from North Africa, the Middle East, and Asia lowered the risk of death from all causes compared with the non-CALD reference group; the risk of death in European backgrounds was similar to that observed in the non-CALD group. This might be because the European group was the oldest among all CALD groups. This suggests that age adjustment could have reversed the pattern observed.

### Suicide risk following self-harm (Sample 2)

Sample 2 data showed that of the 16,928 people, 4.8% died by any cause by 28 March 2018 (*n* = 814), and nearly half (42.3%) of those who died by suicide (*n* = 344). The incidence rate was 3.9 (CI: 3.5–4.3)/1,000 person-years, and it was higher in the non-CALD group than in the CALD group (Tables 2, 4).

**Table 4:** Fifteen leading causes of death among self-harm patients admitted to hospital from 01 Jan 2013 and 31 Dec 2017 (Sample 2).

**Cause of death**	**Non-CALD**		**CALD**		**Total**	
	***n***	***%***	***n***	***%***	***n***	***%***
Intentional self-harm by hanging, strangulation, and suffocation (X70)	146	*20.7*	18	*16.4*	164	*20.1*
Intentional self-poisoning by and exposure to other and unspecified drugs, medicaments and biological substances (X64)	53	*7.5*	8	*7.3*	61	*7.5*
Malignant neoplasm of bronchus and lung unspecified (C349)	‡	‡	†	†	26	*3.2*
Intentional self-poisoning by and exposure to antiepileptic, sedative-hypnotic, antiparkinsonism and psychotropic drugs, not elsewhere classified (X61)	‡	‡	†	†	26	*3.2*
Chronic ischaemic heart disease (I259)	‡	‡	†	†	20	*2.5*
Intentional self-harm by jumping or lying before moving object (X81)	‡	‡	†	†	16	*2.0*
J449 Chronic obstructive pulmonary disease, unspecified	‡	‡	†	†	15	*1.8*
Accidental poisoning by and exposure to narcotics and psychodysleptics [hallucinogens], not elsewhere classified (X42)	‡	‡	†	†	15	*1.8*
Intentional self-harm by sharp object (X78)	‡	‡	†	†	15	*1.8*
Acute myocardial infarction, unspecified (I219)	‡	‡	†	†	12	*1.5*
Accidental poisoning by and exposure to antiepileptic, sedative-hypnotic, antiparkinsonism and psychotropic drugs, not elsewhere classified (X41)	‡	‡	†	†	11	*1.4*
Intentional self-poisoning by and exposure to carbon monoxide and other gases and vapors (X67)	‡	‡	†	†	11	*1.4*
Poisoning by and exposure to other and unspecified drugs, medicaments and biological substances, undetermined intent (Y14)	‡	‡	†	†	11	*1.4*
Other ill-defined and unspecified causes of mortality (R99)	‡	‡	†	†	10	*1.2*
Intentional self-harm by drowning and submersion (X71)	‡	‡	†	†	9	*1.1*
**Total**	**304**	***100.0***	**40**	***100.0***	814	*100.0*

^†^Range was reported instead of the absolute number because cell values were < 6.

^‡^Other cells in the same row and/or column may also be suppressed and reported with “Range ‡” to maintain confidentiality.

Table 4 presents the 15 leading causes of death among the 814 deaths over the follow-up period. Hanging was the first leading cause of death (X70; *n* = 164, 20.7%), followed by self-poisoning (X64, X61; *n* = 87, 10.7%), cancer (C349), and heart disease (I259; Table 4).

Table 5 presents the results of Cox's proportional hazards regression models to examine the risk factors for suicide death after hospital admissions for self-harm. Suicide after self-harm was more common in male individuals, older people, and those living in major cities in Victoria. Suicide was also more common among those who had been self-poisoned by other substances than pharmaceutical poisoning in the index hospital admissions for self-harm. Linkage data showed that suicide risk was increased among self-harm patients with pre-existing mental health conditions (before index self-harm admission) but lower among those who were diagnosed with mental health disorders at the time of the index episode. The risk was not different among patients who had previous self-harm hospital treatment.

**Table 5:** Risk of death due to suicide, Cox's proportional hazards regression models: univariate, partly adjusted and fully adjusted models.

	**Death suicide**					**Model 1**			**Model 2**			**Model 3**			**Model 4**			**Model 5**		
	**Total**	**Death**	**%**	**CI 95%**		**HR**	**95% CI**		**HR**	**95% CI**		**HR**	**95% CI**		**HR**	**95% CI**		**HR**	**95% CI**	
**CALD by regions of birth**																				
**Non-CALD**	**14,625**	**304**	***2.1***	***1.8***	***2.3***	**R**			**R**			**R**			**R**			**R**		
**CALD**	**2,303**	**40**	**1.7**	***1.2***	***2.3***	**0.9**	***0.6***	***1.2***	**0.7** ^§^	***0.5***	***1.0***	**0.70** ^§^	***0.5***	***0.1***	**0.7** ^§^	***0.5***	***0.9***	**0.69** ^§^	***0.5***	***1.0***
Europe^*^	691	24	*3.5*	*2.1*	*4.8*	1.7^§^	1.1	2.6	1.0	*0.7*	*1.6*	1.0	*0.6*	*1.6*	1.0	*0.6*	*1.5*	1.0	*0.6*	*1.5*
Asia	1,030	(10–20)^‡^	^‡^	*0.4*	*1.7*	0.5^§^	0.3	1.0	0.6	*0.3*	*1.0*	0.6	*0.3*	*1.0*	0.5^§^	*0.3*	*1.0*	0.6	*0.3*	*1.0*
Others^**^	582	(0–6)^†^	^†^	*0.1*	*1.6*	0.4	0.2	1.0	0.4^§^	*0.2*	*0.9*	0.4^§^	*0.2*	*0.9*	0.4^§^	*0.2*	*0.9*	0.4^§^	*0.2*	*0.9*
**Sex**																				
**Male**	**6,398**	**194**	***3.0***	***2.6***	***3.5***				**1.9** ^§^	***1.5***	***2.3***	**1.9** ^§^	***1.5***	***2.4***	**1.6** ^§^	***1.3***	***2.0***	**1.6** ^§^	***1.3***	***2.0***
Female	10,530	150	*1.4*						R			R			R			R		
**Age groups**																				
**15–24**	**5,713**	**43**	***0.8***	***0.5***	***1.0***				**R**			**R**			**R**			**R**		
25–34	3,177	60	*1.9*	*1.4*	*2.4*				2.3^§^	*1.6*	*3.5*	2.3^§^	*1.6*	*3.4*	2.2^§^	*1.5*	*3.3*	2.2^§^	*1.5*	*3.3*
35–44	2,968	73	*2.5*	*1.9*	*3.0*				2.8^§^	*1.9*	*4.1*	2.0^§^	*1.9*	*4.1*	2.7^§^	*1.8*	*4.0*	2.6^§^	*1.7*	*3.9*
45–54	2,456	81	*3.3*	*2.6*	*4.0*				3.9^§^	*2.7*	*5.7*	3.8^§^	*2.5*	*5.7*	3.5^§^	*2.3*	*5.2*	3.4^§^	*2.3*	*5.2*
55–64	1,290	32	*2.5*	*1.6*	*3.3*				2.9^§^	*1.8*	*4.6*	2.7^§^	*1.7*	*4.6*	2.6^§^	*1.6*	*4.3*	2.5^§^	*1.5*	*4.2*
65+	1,194	54	*4.5*	*3.3*	*5.7*				5.3^§^	*3.5*	*8.0*	5.1^§^	*3.2*	*8.1*	4.1^§^	*2.5*	*6.8*	4.3^§^	*2.6*	*7.1*
**ARIA**																				
**Major cities of Victoria, Australia**	**10,355**	**202**	***2.0***	***1.7***	***2.2***							**R**			**R**			**R**		
Inner Regional of Victoria, Australia	5,107	125	*2.4*	*2.0*	*2.9*							1.2	*1.0*	*1.6*	1.1	*0.9*	*1.4*	1.1	*0.9*	*1.5*
Outer Regional of Victoria, Australia	1,210	14	*1.2*	*0.6*	*1.8*							0.7	*0.4*	*1.2*	0.6	*0.9*	*0.3*	0.6	*0.3*	*1.0*
**SEIFA**																				
**Decile 1–5 (greater disadvantage)**	4,892	85	*1.7*	*1.4*	*2.1*							0.8	*0.6*	*1.0*	0.8	*0.6*	*1.0*	0.8	*0.6*	*1.0*
Decile 6–10 (greater advantage)	**11,783**	**256**	***2.2***	***1.9***	***2.4***							**R**			**R**			**R**		
**Marital status**																				
**Never married**	**10,067**	**158**	***1.6***	***1.3***	***1.8***							**R**			**R**			**R**		
Widowed/divorced/separated	1,949	63	*3.2*	*2.4*	*4.0*							1.1	*0.8*	*1.6*	1.1	*0.8*	*1.6*	1.1	*0.8*	*1.6*
Currently married/defacto	4,556	121	*2.7*	*2.2*	*3.1*							1.0	*0.8*	*1.4*	1.0	*0.8*	*1.3*	1.1	*0.8*	*1.4*
**Mechanisms of self-harm injury**																				
**Poisoning- pharmaceuticals**	**13,253**	**210**	***1.6***	***1.4***	***1.8***										**R**			**R**		
Poisoning other substances	862	31	*3.6*	*2.4*	*4.8*										1.9^§^	*1.3*	*2.8*	2.0^§^	*1.3*	*2.9*
Sharp object	1,841	35	*1.9*	*1.3*	*2.5*										1.2	*0.8*	*1.8*	1.2	*0.9*	*1.8*
Other means	972	68	*7.0*	*5.4*	*8.6*										4.3^§^	*3.1*	*5.9*	4.5^§^	*3.2*	*6.2*
**Place of self-harm**																				
**Home**	**8,244**	**198**	***2.4***	***2.1***	***2.7***										**R**			**R**		
Health care center	1,032	19	*1.8*	*1.0*	*2.7*										0.7	*0.4*	*1.1*	0.6	*0.4*	*1.0*
Other settings	1,097	30	*2.7*	*1.8*	*3.7*										0.6^§^	*0.4*	*0.9*	0.6^§^	*0.4*	*0.9*
Missing	6,555	97	*1.5*	*1.2*	*1.8*										0.7^§^	*0.5*	*0.9*	0.7^§^	*0.5*	*0.8*
**Length of stay**																				
**< 2 days**	**9,847**	**119**	***1.2***	***1.0***	***1.4***										**R**			**R**		
2–7 days	4,159	140	*3.4*	*2.8*	*3.9*										2.4^§^	*1.8*	*3.1*	2.3^§^	*1.8*	*3.0*
8–30 days	2,214	69	*3.1*	*2.4*	*3.8*										2.0^§^	*1.5*	*2.8*	1.8^§^	*1.3*	*2.5*
31+ days	708	16	*2.3*	*1.2*	*3.4*										0.9^§^	*0.5*	*1.6*	0.8	*0.4*	*1.4*
**Serious injury**																				
Yes	187	19	*10.2*	*5.8*	*14.5*										2.2^§^	*1.3*	*3.6*	2.2^§^	*1.3*	*3.7*
**No**	**16,555**	**321**	***1.9***	***1.7***	***2.1***										**R**			**R**		
**Any comorbidity CHARLSON**																				
Yes	1,576	56	*3.6*	*2.6*	*4.5*										1.2	*0.9*	*1.6*	1.1	*0.8*	*1.6*
**No**	**15,352**	**288**	***1.9***	***1.7***	***2.1***										**R**			**R**		
**Any mental health disorder**																				
Yes	9,365	174	*1.9*	*1.6*	*2.1*										0.5^§^	*0.4*	*0.6*	0.5^§^	*0.4*	*0.6*
**No**	**7,563**	**170**	***2.2***	***1.9***	***2.6***										**R**			**R**		
**Pre-existing health conditions**																				
**Hospital admission due to self-harm**																				
Yes	110	(0–6)^†^	^†^	*0.1*	*7.1*													1.4	*0.5*	*3.7*
**No**	**16,818**	**(300–350)** ^‡^	^‡^	***1.8***	***2.2***													**R**		
**ED presentation due to self-harm (last 12 months)**																				
Yes	1,280	20	*1.6*	*0.9*	*2.2*													0.7	*0.4*	*1.1*
**No**	**15,648**	**324**	***2.1***	***1.8***	***2.3***													**R**		
**Hospital admission due to (all F codes)**																				
Yes	3,935	118	*3.0*	*2.5*	*3.5*													1.5^§^	*1.2*	*2.0*
**No**	**12,993**	**226**	***1.7***	***1.5***	***2.0***													**R**		
**Clinical mental health service contact (CMI-ODS)**																				
Yes	5,003	128	*2.6*	*2.1*	*3.0*													1.3	*1.0*	*1.7*
**No**	**11,925**	**216**	***1.8***	***1.6***	***2.1***													**R**		

Death from suicide was identified by following up the 16,928 self-harm patients (who were admitted to hospital from 01 Jan 2013 and 31 Dec 2017) until death or 28 March 2018.

^*^Persons/POC of those who were born in English-speaking countries were excluded from the relevant CALD groups by ROB, and they were included in the non-CALD group.

^**^Others: Oceania and Antarctica; North Africa and the Middle East, Americas, Sub-Saharan Africa;

^†^Range was reported instead of the absolute number because cell values were < 6.

^‡^Other cells in the same row and/or column may also be suppressed and reported with “Range^‡^” to maintain confidentiality.

^§^P-vaue < 0.05.

#### Comparisons of suicide risks following hospital admissions for self-harm among CALD vs. non-CALD

Compared to the non-CALD group, the CALD group had a lower proportion of people who died by suicide following self-harm [1.7% [CI 1.2–2.3] vs. 2.1% [CI 1.8–2.3], respectively]. The risk of suicide after the first self-harm admission between CALD and non-CALD groups was not different in the univariable model, but it became different in the adjusted models (Model 2 to Model 5). CALD people had a 30% lower suicide mortality risk (Table 5).

Among those who died by suicide, 304 people (88.3%) were from the non-CALD group (of those, 91% were from Australia, 4% from England, and the remainder from New Zealand, Scotland, and South Africa). Among those who died by suicide in the CALD group (*n* = 40), above 50% were from SEE. Due to the low suicide incidence of ROB, we regrouped ROB into three major CALD groups: Europe, Asia, and Other regions. Those from Oceania and Antarctica, North Africa and the Middle East, the Americas, and sub-Saharan Africa had a significantly lower incidence of suicide overall and followed a similar pattern of suicide risk by age, sex, and other characteristics; therefore, they were grouped into “Other regions” (Tables 2, 5).

The univariable model showed that compared to the non-CALD group, suicide risk after self-harm was lower in CALD people; more specifically, by regions of birth, the risk was higher in European backgrounds but lower in Asians and other regions group (than the non-CALD group). However, the fully adjusted model showed that the risk of suicide among self-harm patients from Asian and European backgrounds was not different from that of non-CALD people (Table 5).

The fully adjusted models showed similar results to the age and sex-adjusted models, which means the differences in suicide and all-cause mortality risks in different cultural backgrounds were affected mostly by age and sex.

## Discussions

The present study compared the risks of all-cause mortality and suicide following hospitalization for hospital admissions for self-harm between people from CALD and non-CALD backgrounds. Overall, the study found that CALD people had a lower risk of all-cause mortality and suicide than the non-CALD group. By regions of birth (ROB), those from North Africa, the Middle East, and Asia had lower all-cause mortality risk compared with the non-CALD reference group, but suicide risk following self-harm was not different between Asian and non-CALD backgrounds. The results suggest that the risks of all-cause mortality and suicide vary among different cultural backgrounds grouped by region of birth.

One of the most important findings of this study was that, compared to non-CALD people, some CALD groups by ROB were at lower risk of death and suicide following self-harm; none of the CALD backgrounds (based on regions of birth) was associated with a higher risk than the non-CALD groups, after adjusting for age and sex. This is consistent with earlier research in England (13), revealing that Black people (suicide proportion: 0.78%) had a lower risk of suicide following self-harm Emergency Department presentation than white self-harm patients (2.1%; *p* = 0.05). We acknowledge that the lower risk of suicide after hospital admissions for self-harm in the CALD group overall (compared with the non-CALD group) is somewhat contrary to common public discourse and expectations around migrant difficulties (12). While the common knowledge was mostly based on the fact that migrants and refugees might be at higher risk of migration stress, language barriers, social isolation, service access barriers, transgenerational trauma, and other risk factors for suicide, self-harm, and mental illnesses, we argued that CALD-related potential protective factors might play an important role in preventing CALD people from suicide death (26). Potential protective factors include healthy migrant effects(32); being well-educated and from a wealthy background (this can be the case for some international students and skilled migrants entering Australia); and migration can act as a healthy (and in some cases, wealthy) selection process. Furthermore, some CALD people might be protected by specific cultural or spiritual beliefs about the body (e.g., the Confucian principle that their bodies belong to their parents and they should not harm the body). Participating in religious activities is also found to be a protective factor against suicide. These protective factors for suicide might explain some of the lower risk of suicide following hospital admissions for self-harm among CALD people. The healthy migrant effect specifically can explain the lower rates of death in the CALD (vs. non-CALD) group. We suggest future qualitative studies to explore protective and risk factors for self-harm and suicide among CALD people. The qualitative results might inform clinical decisions following a psychological assessment. If possible, some possible *protective factors* could be considered in developing suicide prevention strategies; potentially, these could inform prevention in the non-CALD group, which was at higher risk for fatal outcomes in our study and others (13).

Self-harm in CALD people may be underestimated in the results presented here. This study focussed on examining the risk of suicide after hospital admissions for self-harm, noting that some CALD people might avoid accessing hospital services due to factors such as stigma and language barriers unless they had serious injuries requiring immediate care. Also, CALD people (in particular new migrants) might be less likely to have family or friends with them after self-harm to bring them to the hospital (compared with non-CALD). Fatal outcomes in CALD people may also be underestimated: suicide is considered to be a sin in some cultures, and therefore suicide might be masked as other injuries such as unintentionally falling from height (rather than intentionally) in some CALD populations. Those possibilities might have affected the result of the study; further research is needed to test these possibilities. An investigation of health outcomes following self-harm in the community (not limited to hospital-admitted participants) is recommended.

To the best of our knowledge, there are very little data on suicide and self-harm in CALD communities in Australia that could be used to compare the findings. This might be due to a lack of accurate, reliable data on suicide in CALD populations. According to Bowden et al. (33), data collection in these communities is inconsistent and unreliable, resulting in either limited data availability or none at all on suicide deaths and suicidality among these communities (33). We suggest more attention should be paid to strengthen self-harm and suicide data quality and coverage. This could include the development (or improvement) of self-harm databases to collect more detailed data on CALD people, capturing information on how people entered Australia and under what circumstances (as refugees or as planned migrants), the main language spoken at home, race, as well as other factors that might be relevant to self-harm, such as family violence and substance use.

Another important finding of this study is that when grouping CALD people by region of birth, there was considerable variation in risks, suggesting that suicide prevention efforts targeting specific cultural groups would be beneficial.

First, of all CALD groups, people from North Africa and the Middle East groups emerged to have lower risks of both all-cause mortality and suicide death. While there are potential cultural heritages that have been shown to play a protective role against suicide, such as religious activity, it might also be a bias because suicide can be intentionally masked or denied in this group if it conflicts with religious beliefs (34, 35). As we also found a lower risk of all-cause mortality in this group compared to the non-CALD group, this suggests underreporting of suicide deaths is less likely, which is the explanation for the lower suicide risk seen in North Africa and the Middle East groups. However, there is still a possibility that self-harm patients might be missed from this group due to underreporting hospital admissions for self-harm. Interventions to increase access to health services as well as data quality improvement should be prioritized, especially for this group.

Second, the risk of suicide following self-harm among those from Asian backgrounds was as high as in non-CALD people. This finding is consistent with the research in England (13), revealing that the risk of suicide following hospital admissions for self-harm in South Asian patients (suicide proportion: 0.93%) and white people (2.1%) was not statistically different (*p* = 0.05). However, provided that in a previous study, we found Asians had a lower rate of hospital-admitted self-harm than the non-CALD group (26), we believe that understanding the reasons why the risk of suicide after self-harm in the Asian group did not statistically differ from the non-CALD group could provide valuable insights into suicide prevention initiatives. Hospital presentation for self-harm is an opportunity for psychological management and other self-harm interventions (36), and self-harm patients who fail to access mental healthcare after self-harm might be placed at higher risk of suicide.

In another data linkage study investigating the likelihood of accessing mental health services after self-harm in CALD communities, we also found that Asian inpatients were less likely to contact mental health services after discharge (18). Thus, a plausible explanation for why the risk of suicide after self-harm in Asians did not differ from the non-CALD group (while other CALD groups by regions of birth had a lower risk) might be related to the lower uptake of mental health services after self-harm associated with some Asian backgrounds (18, 37). Secondary prevention should therefore be augmented for Asian as well as for non-CALD patients. The following section relates to the CALD group: further research specifically in the non-CALD group is highly recommended to inform primary and secondary prevention; this focus falls outside the scope of this study.

To find opportunities for suicide prevention in the group of Asian patients who have self-harmed, we need to understand cultural barriers that prevent the group from contacting mental health services after being discharged from the hospital due to self-harm. Possible barriers include low recognition of mental issues (mental health might be less likely to be discussed in Asian families) and stigma and shame around self-harm and mental health (37, 38). Also, for newly migrated people, poor awareness of mental health and available services could be a barrier, or some might choose to use alternative medicine (39, 40).

Therefore, clearly, there is still scope for suicide prevention in the CALD group by improving the uptake of mental health services following self-harm, especially by people from Asian backgrounds. Because mental health is not commonly discussed within Asian cultures, one of the possible interventions may be to improve awareness of mental health service provisions within their local communities (39, 40), especially for new migrants. Interventions to encourage self-harm patients to access mental health services after discharge from the hospital should also be emphasized (i.e., introducing available psychological services in local areas, referring patients to local mental health services, and informing patients' medical providers regarding their conditions and possible risks for follow-up and further assessment). This sort of information could be provided to patients and their families at the time of discharge (5, 41). Tailored assessments of mental illnesses among those with a history of self-harm should also be considered to better recognize self-harm patients at risk of suicide, especially at primary health service centers. More importantly, due to the possible challenges of discrimination and health inequalities experienced by some CALD people, it is important to consider interventions that are related to the appropriateness and cultural safety of the services themselves (42). Another intervention may be to offer language-specific information, in addition to an anonymous confidential telephone helpline for people to access that could also be language-specific. These suggestions could help inform suicide prevention strategies for CALD communities.

### Limitations

There is potential selection bias in this study, which is limited to hospital-admitted self-harm. First, self-harm admissions do not capture all self-harm; some are not reported to health services, and some are reported to general practitioners. Second, self-harm could be presented as an unintentional injury due to socio-cultural factors such as shame, religious beliefs/mores, and stigma related to self-harm or mental health, especially among people from some CALD backgrounds (43, 44). Third, some self-harm could be left untreated due to shame and stigma and avoidance of healthcare for that reason. Therefore, the overall incidence of self-harm and health service use observed here may underestimate the extent of the problem, and the results should be interpreted with caution.

There are limitations to using only the country of birth to define CALD backgrounds. As presented in the research methodology, the inclusion of Indigenous people in the non-CALD group (because they were mostly born in English-speaking countries) may have increased the incidence of self-harm in the non-CALD group. This is likely to lower the sensitivity of the analysis in being able to distinguish CALD/non-CALD differences; therefore, the findings in this paper may present an underestimate of the underlying differences in outcomes between these groups. Next, comprehensive data on whether people in CALD groups arrived in Australia through refugee status was unavailable, and this factor should be examined when the data is available. Finally, the findings might also be influenced by the length of stay in Australia of migrants and acculturation: higher levels of acculturation appear to increase the risks of lifetime self-harm (45). In addition, some people in the non-CALD group were born overseas in English-speaking countries, but they might culturally be quite distinct from the remainder of the non-CALD group. The next stage of research should examine self-harm differences in comparison groups of CALD, Indigenous people, and non-CALD people with consideration of the impacts of length of stay in Australia and acculturation.

Moreover, as CALD individuals are migrants, there might be a possibility that some CALD individuals have migrated again, meaning a loss of follow-up in mortality databases. Future research is needed in which immigration linkage data would be involved.

Additionally, to compare the risk of suicide among people from various regions of birth, we grouped regions into bigger groups. Although they showed some similar patterns, we acknowledge that these groups are heterogeneous, and this might affect the results.

### Implications

Further research is needed to build on this result, but tentatively, the findings suggest that following hospital-admitted self-harm, being from a CALD background was associated with a lower risk of death and suicide; the risk varied by region of birth. The risk of suicide after self-harm admission among those from Asian backgrounds was as high as that of those from non-CALD backgrounds, while it was lower among people from North Africa and the Middle East. The findings of this study demonstrate the need for suicide prevention following hospital admissions for self-harm for non-CALD people (who are at greatest risk overall) as well as for specific CALD groups (the focus of this study) to save lives and reduce the overall rate of suicide in Australia. To prevent suicide following hospital admissions for self-harm in CALD people, different strategies and resource allocation are likely to be needed for different CALD populations because of their different risk levels and possible risk/protective factors. Qualitative data to understand the risk and protective factors for suicide after self-harm in CALD communities could help inform clinical decisions following psychological assessment. Interventions should focus on raising awareness about the availability of health and mental health services as well as improving health equality in seeking healthcare, especially for new Asian migrants or refugees. More attention should be paid to improving the existing self-harm surveillance systems and on the reporting and publication of CALD-specific self-harm and suicide data.

## Data availability statement

The linkage data might contain sensitive information about self-harm and suicide. The data will not be shared for confidentiality reasons. Requests to access these datasets should be directed to https://vahi.vic.gov.au/ourwork/data-linkage.

## Ethics statement

The studies involving humans were approved by Monash University Human Research Ethics Committee. The studies were conducted in accordance with the local legislation and institutional requirements. Written informed consent for participation was not required from the participants or the participants' legal guardians/next of kin in accordance with the national legislation and institutional requirements.

## Author contributions

TP: Conceptualization, Data curation, Formal analysis, Investigation, Methodology, Project administration, Visualization, Writing – original draft, Writing – review & editing. KO'B: Conceptualization, Supervision, Writing – review & editing. SL: Conceptualization, Supervision, Writing – review & editing. KG: Conceptualization, Supervision, Writing – review & editing. JB-G: Conceptualization, Supervision, Writing – review & editing, Data curation, Methodology.

## Footnotes

^1^Participants from English-speaking backgrounds were excluded from the relevant CALD groups by ROB, and they were included in the non-CALD group.
